# The Role of Phosphoinositide 3-Kinase Signaling in Intestinal Inflammation

**DOI:** 10.1155/2012/358476

**Published:** 2012-04-09

**Authors:** Catherine M. Cahill, Jack T. Rogers, W. Allan Walker

**Affiliations:** ^1^Mucosal Immunology Laboratory, Department of Pediatrics, 114 16th Street, Massachusetts General Hospital for Children, Charlestown MA 02129, USA; ^2^Neurochemistry Laboratory, Department of Psychiatry, 149 13th Street, Massachusetts General Hospital, Charlestown MA 02129, USA

## Abstract

The phosphatidylinositol 3-kinase signaling pathway plays a central role in regulating the host inflammatory response. The net effect can either be pro- or anti-inflammatory depending on the system and cellular context studied. This paper focuses on phosphatidylinositol 3-kinase signaling in innate and adaptive immune cells of the intestinal mucosa. The role of phosphatidylinositol 3-kinase signaling in mouse models of inflammatory bowel disease is also discussed. With the development of new isoform specific inhibitors, we are beginning to understand the specific role of this complex pathway, in particular the role of the *γ* isoform in intestinal inflammation. Continued research on this complex pathway will enhance our understanding of its role and provide rationale for the design of new approaches to intervention in chronic inflammatory conditions such as inflammatory bowel disease.

## 1. Phosphatidylinositol 3-Kinase Family

 The phosphatidylinositol 3-kinases (PI3-K) are a family of lipid kinases involved in a broad range of cellular responses from cell cycle regulation, apoptosis, growth, and cell survival, making this a highly complex signaling network involved in cellular homeostasis [1]. Dysregulation of this complex pathway can lead to diseases such as cancer, inflammation, and autoimmunity, all associated with inflammatory bowel disease. 

 Phosphatidylinositol 3-kinases (PI3-K) phosphorylate the D-3, OH position of the inositol head groups of phosphoinositide lipids, phosphatidylinositol (PtdIns), phosphatidylinositol (4)-phosphate (PtdIns(4) P), and phosphatidylinositol (4,5)-biphosphate (PtdIns(4,5) P2) [2]. This results in the formation of PtdIns PIP, PtdIns(3,4) PIP2, and PtdIns(3,4,5) PIP3, respectively. These lipids bind to the pleeckstrin homology domains (PH) of proteins, thereby controlling the activity and subcellular localization of a variety of signal transduction molecules. PI3-kinases can be divided into 3 main classes on the basis of their *in vitro* lipid substrate specificity. 

### 1.1. Class 1

**Figure 1:**
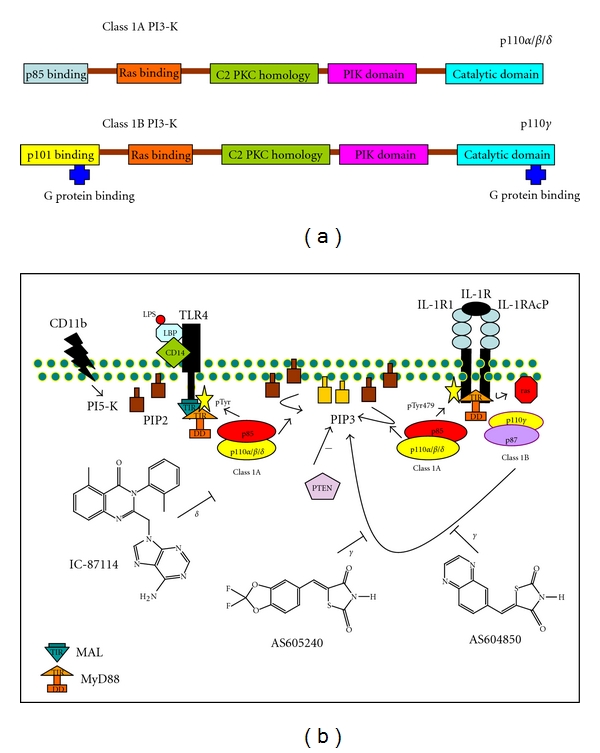
(a) domain structure of the catalytic subunits of the class 1 PI3-Kinases. Three genes PIK2CA, PIK3CB, and PIK3CD code for the class 1A, p110*α*, *β*, and *δ* isoforms of PI3-K. They have an N-terminal p85 binding domain, a C-terminal catalytic domain, a ras binding domain, a C2 (PKC homology domain), and a phosphatidylinositol kinase homology (PIK) domain. Class 1B is a heterodimer composed of either a p101 or PIKAP (PI3-K*γ* adapter protein of 87 kDa) regulatory subunit and a catalytic p110*γ* subunit. GPCRs activate PI3-K*γ* through interactions with G*βγ* [9]. The catalytic p110*γ* subunit has significant sequence homology to class 1A catalytic subunits; however, its regulatory subunits, p101 and p87, are different from p85. (b) class 1A and class 1B phosphatidylinositol 3-kinases are activated downstream of toll/IL-1 receptors in myeloid cells, and selective isoform-specific inhibitors have been developed. Binding of LPS to CD14 likely induces PI5-kinase to generate PIP2 downstream of integrin *β*2 (CD11) signaling [10]. LPS/CD14 interaction regulates steady state levels of PIP2 at the plasma membrane and the localization of the MAL adaptor protein. MAL facilitates the TIR-mediated recruitment of the MyD88 adaptor. Tyrosine phosphorylation by a src-related kinase on the TIR domain of MAL/MyD88 or other TLR4 adaptor serves to recruit SH2 containing protein p85, the PI3-K regulatory sub-unit. The catalytic subunits of PI3-K, p110, *α*, *β* and *δ* and *γ* isoforms mediate the phosphorylation of PIP2 to PIP3. Downstream of the IL-1 receptor, a ras-dependent pathway to the activation of class 1B, PI3-Kinase *γ* isoform, has recently been reported, associated with myeloid cell trafficking tumor growth and progression [8]. IC-87114 is the first selective PI3-K*δ* inhibitor. This selectivity was unexpected given that the residues that line the ATP binding pocket of class 1 PI3-Ks are highly conserved. AS-604850 and AS-605240 are selective, ATP-competitive inhibitors of the PI3-K*γ* isoform shown to inhibit intestinal inflammation in murine colitis models.

The Class 1 PI3-Ks are a major focus of study as it is these isoforms that are coupled to extracellular stimuli [3]. The **class 1A** enzymes encode 5 regulatory subunits encoded by 3 separate genes, PIK3r1 encoding p85*α* and alternative transcripts, p50 and p55, PIK3r2, encoding p85*β* and PIK3r3 encoding p55*γ*. These regulatory subunits each pair with one of the class 1 catalytic subunits, p110*α*, p110*β* and p110*δ* (Figure 1(a)). 

 The regulatory subunits function to recruit the complex to the plasma membrane following receptor ligation. The interaction between p85 and the receptor complex is mediated by a high-affinity interaction between the p85 Src homology 2 (SH2) domain and the specific tyrosine-phosphorylated sequences within the cytoplasmic tail of the receptor. The process recruits the p110 catalytic domain to the plasma membrane where it phosphorylates its main substrate PtdIns(4,5) P2 to generate PtdIns(3,4,5) P3. It has recently been demonstrated that p85 itself is regulated by phosphorylation, and this determines its ability to associate with p110 [4]. Recruitment to the plasma membrane *via* association of p85 with signaling complexes containing Shc, Grb2, and Gab2 in response to cytokines such as interleukin-1 (IL-1) has also been reported [5]. The catalytic subunit, p110, also binds to activated ras which might also stabilize association with the plasma membrane after recruitment to the receptor complex by p85. Class 1A isoforms are activated downstream of T-cell receptors (TCRs) B-cell receptors (BCR) and costimulatory receptors as well as cytokine receptors that are phosphorylated by tyrosine kinases after receptor engagement with ligand (Figure 1(a)). Class 1A PI3-Ks have been proposed to act as negative regulators downstream of toll-like receptor (TLR)-induced signaling thereby affecting IL-12 production by dendritic cells (DCs) [6, 7]. Thus, inhibition of PI3-Ks could upset the balance of Th1/Th2 responses. The **class 1B** isoform p110*γ* associates with one of 2 regulatory subunits, p84/87 or p101 [5]. Until recently it was thought that this class was downstream specifically of G-protein-coupled receptor *βγ* subunits. However, it has now been demonstrated that p110*γ*/p87 is also downstream of toll-like receptors/IL-1 receptors in myeloid cells making it a convergent point controlling tumor inflammation and progression [8] (Figure 1(b)).

### 1.2. Class 2

These PI3-Ks, encompassing PI3-kinase-C2*α*, C2*β* and C3*γ* are characterized by a C2 domain that mediate calcium/lipid binding in protein kinase C isoforms. Class 2 PI3-Ks use (PtdIns(4) P) as their preferred substrate. Class 2 PI3-Ks have not been isolated in association with a regulatory subunit. This class binds to clathrin and their localization to coated pits suggests a role in membrane trafficking and receptor internalization [11].

### 1.3. Class 3

These PI3-Ks utilize only PtdIns as substrate thus creating PtdIns(3) P. In mammalian cells, this kinase is involved in the movement of proteins through the lysosome [11]. The mechanism of activation of classes 2 and 3 PI3-Ks *in vivo* is not fully understood as is their role in the immune system.

## 2. Tissue Distribution, Feedback Regulation, and Pharmacological Inhibition

 While PI3-K*α* and *β* have a broad tissue distribution, PI3-K*δ* and *γ* are predominantly expressed in leukocytes. PI3-K*δ* is also expressed in neurons and in some cancers such as breast and melanoma, while PI3-K*γ* is also expressed in endothelium and heart. There has been much interest in the PI3-k*δ* and *γ* isoforms as they represent promising targets for selective inhibition of PI3-K in inflammatory and autoimmune conditions [12]. Although there is evidence suggesting that PI3-K*δ* and *γ* act in partnership, there is also evidence that they play complimentary roles in the immune system. Murine knockouts of the p110*α* and *β* genes results in embryonic lethality with reports suggesting that p110*α* plays a role in cell survival and p110*β* isoform being important in cell proliferation. A mouse harboring mutation in the p110*δ* isoform (D910A/D910A), although viable and fertile, demonstrates B- and T-cell defects including improper maturation, defective antigen receptor signaling, and impaired humoral immune responses with a shift towards Th2 responses. These mice develop chronic segmental colonic inflammation [12].

 The PI3-K*δ* isoform is crucial to the function of CD4+CD25+FoxP3+ Treg cells which produce the anti-inflammatory cytokine, IL-10. Using PI3-Kinase D910A^−/−^ mice, it was shown that PI3-K*δ* plays a key role in Treg-mediated suppression of CD4+CD25−T-cell proliferation and inflammation. Mice expressing kinase inactive PI3-K*δ* develop a mild inflammatory bowel disease phenotype which might be indicative of such a suppressive mechanism [13]. The serious defects in immune development in double knockout PI3-K*γδ* ^−/−^ mice prevent a detailed understanding of the selective roles of these subunits. Knockout of the p110*γ* isoform in mice suggests that this isoform is critical for full B- and T-cell antigen receptor signaling [14].

 Negative feedback regulation of PI3-K signaling by the 3′ phosphatase PTEN (phosphatase and tensin homolog) and the 5′ phosphatase SHIP1 (SH2-domain containing inositol-5-phosphatase 1) and SHIP2 is essential to control constitutive activation and associated disease such as cancer. PTEN is a tumor suppressor mutated or deleted in a variety of tumors. Cells lacking PTEN have elevated levels of PtdIns(3,4) P2 and PtdIns(3,4,5) P3 with constitutive activation of PI3-K. SHIP has an important role in lymphocytes with loss of SHIP culminating in the development of autoimmunity. PTEN and SHP2 are ubiquitously expressed while SHP2 is mainly restricted to leukocytes [2].

 As PI3-K has a function in normal immune homeostasis, complete blockade of PI3-K activity may compromise immunity and increase susceptibility to infections particularly during chronic inflammation. The first generation PI3-K inhibitors, wortmannin, and LY294002 are unsuitable for therapeutic use because of the lack of stability and selectivity or because of toxicity issues. As there is a high degree of amino acid sequence homology between the ATP-amino acid binding pockets of the four class 1 PI3-Ks, the search for selective small molecule isoform specific PI3-K inhibitors was challenging. The discovery of the quinazolinone purine series of inhibitors by ICOS Corp with IC-87114, demonstrating selective PI3-K*δ* inhibition with negligible potency against PI3-K*α* and *β* isoforms was an important advance. Selective inhibition of PI3-K*γ* has also been accomplished by Merck Serono S.A. with AS-605240 and AS-604850 [12] (Figure 1(b)). Dual specificity, PI3-K*γ* and *δ*, has been accomplished with TG-100-115 from TargeGen. Pharmacological inhibition of PI3-K*γ* which is involved in immune effector cell recruitment may reduce immune surveillance. Therefore, caution should be taken when using selective PI3-K*γ* inhibitors as they could also potentially interfere with its nonimmune function, for instance its' involvement in cardiac contractility. Recent evidence that PI3-K*β* and *γ* can couple to the same GPCRs in a redundant manner may further limit the usefulness of these inhibitors in the immune system.

## 3. PI3-Kinase, Recruitment by IL-1, and TLR Family Receptors

Innate immune responses are triggered through toll-like receptors (TLRs) that recognize a variety of microbial antigens called pattern-associated molecular patterns (PAMPs). The extracellular region contains leucine-rich repeat (LRRs) domains specialized to recognize a specific microbial ligand. TLRs and IL-1 receptors have in common a TIR domain (toll/IL-1 receptor). The toll/Interleukin-1 receptor (TIR) domain is the conserved intracellular domain of the two families of receptors and is also shared by the downstream adapter molecule MyD88. Upon receptor activation, it is believed that a TIR domain signaling complex is formed between the receptor and the adapter and is responsible for mediating the downstream signaling generated by the engagement between TLRs and the PAMPs (Figure 1(b)) [15]. In humans, 10 TLRs have been identified. We will focus here on TLR4 and TLR5 which are the receptors for bacterial lipopolysaccharide, LPS and the lipoproteins, flagellin, respectively. These TLRs reside on the plasma membrane [16, 17]. Class 1A and class 3 PI3-Ks have been shown to play a role in TLR signaling [18]. Once activated PI3-K regulates TLR signaling in both positive and negative ways. PI3-K is believed to be a gate-keeper to control excessive innate immune responses and is an early event in TLR signaling.

### 3.1. The Adaptor Proteins MyD88 and Mal Are Involved in PI3-Kinase Recruitment by TLRs

**Figure 2:**
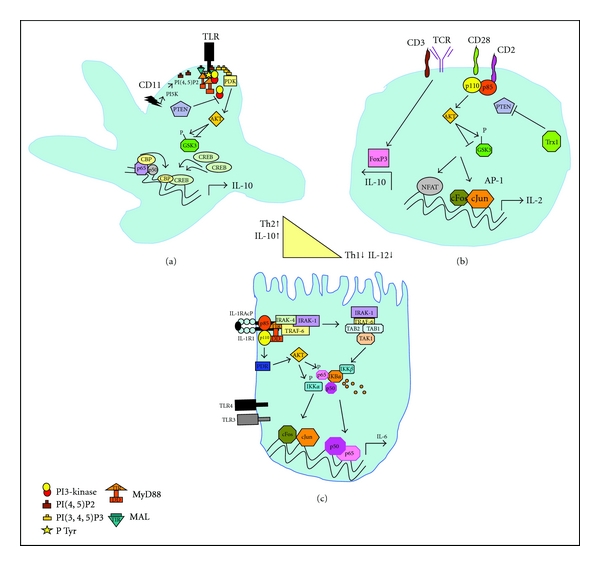
(a). PI3-kinase/AKT/GSK3*β* pathway control of pro- and anti-inflammatory cytokine production in innate immune cells determines the balance of Th1 and Th2 immune responses. Plextrin homology (PH) domain containing kinases, PDK, and AKT are recruited to the plasma membrane and bind to PIP3. PDK phosphorylates AKT on Thr308 in the activation loop, and this is followed by Ser473 phosphorylation. For MyD88-dependent signaling, TLR-mediated inhibition of GSK3, *via* AKT phosphorylation of its Ser 9 residue leads to increases in DNA binding of cAmp response element binding protein 1 (CREB), which displaces the coactivator CBP from NF*κ*B. The increased CREB activity leads to production of the anti-inflammatory cytokine IL-10 (Th2 cytokine) and lowered IL-12 production. Inhibition of PI3-K via dephosphorylation of PIP3 by the phosphatase PTEN enables GSK3 to remain active to inhibit transcription factors such as cJun and CREB thereby decreasing IL-10, increasing NF*κ*B-mediated IL-12 expression, and enhancing Th1 responses. (b) lamina propria T (LPT) cells are hyporesponsive to TCR stimulation and use the alternative CD-2 pathway. PI3-kinase AKT/GSK3*β* pathway downstream of CD-2 likely targets the AP-1 and NFAT sites on the IL-2 promoter. The activity of the PIP3 phosphatase, PTEN is likely reduced in LPT cells due to the increased thioredoxin (TrX1) in these cells. Multiple TCR stimulation of LPT cells has been reported to induce FOXP3/IL-10 producing immunosuppressive Treg cells [21]. (c) PI3-kinase-dependent pathways to IL-6 gene transcription in response to IL-1 in Caco-2 intestinal epithelial cells. IL-1 binding to the IL-1R1 increases its affinity for the co-receptor, the IL-1 receptor accessory protein (IL-1RAcP). Formation of the signaling module containing the MyD88 adaptor protein together with phosphorylated IRAK (interleukin-1 receptor-associated kinase) and TRAF-6 (TNF receptor-associated factor) is essential for PI3-K recruitment and AKT activation. The TAK1 (TGF*β* activated kinase) signaling module is likely a separate parallel pathway to NF*κ*B activation. We identified 2 separate pathways to the induction of IL-6 transcription in response to IL-1, the first is a novel IKK*α*-dependent pathway involving phosphorylation of the T23 residue on IKK*α*, upstream of AP-1 (activator protein 1) activation, and the second is an AKT-dependent activation of NF*κ*B, likely via phosphorylation of the p65 subunit [22].

TLR signaling pathways have been studied extensively in the context of antigen presenting cell (APC) function. All TLRs except TLR3 (ligand for dsRNA) mediate signals through a pathway *via *the TIR domain containing adaptor MyD88. MyD88 mediates TLR signaling through 2 critical domains, the TIR domain recruits MyD88 to the TLR after engagement and the MyD88 death domain (DD) couples TLR:MyD88 association to the activation of downstream targets associated with inflammation. The cytosolic domains of TLRs2, 3, and 5 all bear a conserved YXXM, PI3-K consensus binding site. A recent study demonstrated however that there was no such domain present on the TLR4/LPS receptor, leaving open the question whether the SH2-mediated association of p85 to TIR family members is the only way of activating PI3-Kinase [19]. As MyD88 is one of 4 adaptors that binds to TLR4 and it has been reported that PI3-K mediated activation of NF*κ*B depends on the MyD88 TIR domain and on the IRAK1 DD death domain, it is likely that p85 binds to the MyD88 TIR domain in response to TLR4 ligation [20] (Figures 1(b) and 2(a)).

Alignment of MyD88 TIR domains of several vertebrate species reveals a highly phylogenetically conserved putative SH2, YKXXM motif which was shown to promote PI3-K recruitment in response to TLR9 stimulation [19]. Interestingly, a dominant negative mutant of MAL (MyD88 adaptor-like protein) had no effect on either IL-1 or LPS activation of AKT [20]. More recently, it has been demonstrated that TIR-containing Mal also directly interacts with the regulatory subunit of PI3 kinase, p85*α*, and that Mal-p85*α* interaction drives PI3K-dependent phosphorylation of Akt, PIP3 generation, and macrophage polarization [23].

### 3.2. PI3-Kinase Recruitment to the IL-1R Depends on MyD88, IL-1RAcP, and IRAK

Interleukin-1 receptors are transmembrane glycoproteins which lack a catalytic domain. IL-1R therefore recruits the serine/threonine kinase, interleukin-receptor-associated kinase, IRAK. The C-terminal portion of the IL-1R is essential for IL-1 signaling and therefore interacts with accessory signaling components. IL-1 stimulation induces aggregation of the IL-1R1 with the IL-1 receptor accessory protein (IL-1RAcP) which increases the binding affinity of IL-1R [24]. The activated IL-1RAcP complex then recruits IRAK through binding to its cytoplasmic tail. MyD88 is the adaptor protein that is involved in IL-1R and toll-like receptor induction of NF*κ*B and JNK. By directly binding IRAK-1 and IRAK-4, MyD88 serves as a bridging protein enabling IRAK-4-induced phosphorylation of IRAK-1 (Figures 1(b) and 2(c)).

A highly conserved consensus binding site for PI-3 kinase is present on the cytoplasmic domain of IL-1R (Tyr-E-X-Met). The IL-1 receptor is tyrosine-phosphorylated in response to IL-1 stimulation, and it was shown that, Tyr479 was essential for PI3-kinase recruitment and activation [25]. Interestingly Tyr479 phosphorylation was also shown to be upstream of NF*κ*B activation. Both the N- and C-terminal SH2 domains of p85 can bind the IL-1R. It was later determined that the C-terminus of the IL-1RAcP also binds p85 [26, 27]. The IL-1RAcP as well as MyD88 have similar consensus binding sites for PI3 kinase. Although the IL-1LRAcP does contain a C-terminal TIR domain, this does not appear to be tyrosine-phosphorylated in response to IL-1 [28]. It was later demonstrated that the terminal 26 aa of IL-1RacP was essential for PI3-Kinase recruitment and activaton of NF*κ*B but had no effect on activation of JNK/SAPK in response to IL-1 [27]. Reddy et al. demonstrated that PI3-K was activated by interleukin-1 and that IL-1 receptor activation induced the association between the type 1 receptor and the p85 regulatory subunit [29]. Further, wortmannin and a dominant negative p85 subunit inhibited IL-1 activation of both NF*κ*B and AP-1.

The binding of IL-1 to the type 1 IL-1 receptor induces cascades of intracellular events including activation of mitogen-activated protein kinases (MAPKs) involved in the activation of AP-1 and I*κ*B kinases (IKKs) involved in the activation of NF-*κ*B [24]. Activation of PI-3 kinase, by IL-1, is sufficient for full activation of AP-1 but not NF*κ*B (Figure 1) [29].

Both IL-1R and TLRs activate the central MyD88-IRAK-TRAF6 signaling module. Although PI3-Kinase can bind directly to the IL-1R, it has been shown using ODN oligonucleotides as well as in IRAK1 deficient cell lines that IL-1 activation of PI3-K also depends on IRAK1 and 2 suggesting its involvement in the signaling modules [30]. Whereas IRAK1 appears to interact directly with the IL-1RAcP, IRAK-2 preferentially associates with the IL-1R [17]. To our knowledge no protein-protein interaction between PI3-K and IRAK-1/2 has been reported and TRAF 6-mediated PI3-K function is thought to be indirect *via* its association with the tyrosine kinase Src [31]. In IRAK1 deficient mouse embryo fibroblasts, neither IL-1 nor LPS induced AKT phosphorylation or IL-6 gene induction, and reintroduction of IRAK-1 rendered these cells fully responsive.

## 4. Role of PI3-Kinase Signaling Downstream of IL-1R, TLRs, and TCR Costimulatory Molecules

The coordinated response of innate and adaptive immune cells together with intestinal epithelial cells to luminal commensal and pathogenic bacteria can result in dysregulation of homeostasis resulting in inflammatory bowel disease. This section discusses what is known about PI3-K signaling downstream of these receptors in the relevant cell types.

### 4.1. Role of PI3-K Signaling in Innate Immune Cells

#### 4.1.1. Dendritic Cells

Dendritic cells are important mediators of the innate immune response in the intestine. Dendritic cells have a rich supply of pattern-recognition receptors and are present within Peyers Patch and throughout the lamina propria, producing extensions into the gut lumen to sample luminal antigens. Human intestinal lamina propria DCs express the MHC II marker HLA-DR. This lineage is largely conventional CD11c+ myeloid DCs [32]. Most of the data on intestinal dendritic cells come from mouse models. Recently, a few reports have appeared on human intestinal dendritic cells [32]. Numerous studies have demonstrated altered DC phenotype and function in inflammatory bowel disease (IBD). DCs are more activated and express increased levels of the maturation markers and TLRs as well as producing proinflammatory cytokines [32, 33]. Treatment of ulcerative colitis patients with probiotics in combination with corticosteroids induced a more favorable phenotype with DC producing less inflammatory cytokines and lower TLR expression [33].

PI3-Ks are activated in DCs by many stimuli, including LPS, CpG-oligodeoxynucleotide, many of which induce IL-12. In DCs, PI3-K inhibits p38 Map Kinase which is essential for transcriptional activation of IL-12. Interleukin-12 determines the balance between Th1 cellular-mediated immunity and Th2 humoral, antibody-mediated immunity (Figure 2(a)). Elevated IL-12 will skew towards a Th1 response [7]. Dendritic cells (DCs) are integral to the differentiation of T-helper cells into T-helper type 1 Th1, Th2, and Th17 subsets. Interleukin-6 (IL-6) plays an important part in regulating these three arms of the immune response by limiting Th1 response and promoting Th2 and Th17 responses.

Dendritic cells isolated from intestinal biopsies from patients with inflammatory bowel disease have elevated numbers of TLRs and secreted elevated cytokines. Lamina propria DCs from inflamed human tissue produce higher levels of IL-12, IL-23, and IL-10. Both shared and specific TLR-mediated pathways exist. Shared pathways involve MyD88, Tollip, IRAK, and TRAF6 with the other TLR adaptors, TRIF, and MAL initiating more specific pathways [32]. Thus, TLRs can translate the information regarding the nature of the pathogens into differential cytokine production, thereby polarizing the immune response [6].

Increased IL-6 expression was observed in colon tissues of DC-depleted mice, as well as a more severe colitis when exposed to dextran sodium sulfate (DSS) compared to normal mice, demonstrating that regulation of IL-6 production may contribute to DC-mediated control of intestinal inflammation [34]. Recently, a novel PI3-K dependent pathway to IL-6 production in CD11c DCs was reported involving cKit [35]. Dendritic cells generated from mice expressing a catalytically inactive form of the p110**δ** subunit of phosphatidylinositol3 (PI3) kinase (p110D910A) secreted lower amounts of IL-6 upon stimulation with cholera toxin. These results demonstrate the importance of the c-Kit-PI3 kinase-IL-6 signaling axis in DCs in regulating T-cell responses. Intestinal epithelial cells in close proximity with mucosal DC can influence localization of DC subsets thus conferring mucosal DC specialization. Intestinal epithelial cells produce thymic stromal lymphopoietin (TSLP) which inhibits IL-12 production by DCs in response to bacteria, thus promoting Th2 responses [36].

#### 4.1.2. Intestinal Macrophages

 Resident lamina propria macrophages are unique for their capacity to phagocytose and digest microorganisms without an inflammatory response. Intestinal macrophages are downregulated at both mRNA and protein levels for multiple innate response molecules including the receptors for LPS (CD14) [37]. Intestinal macrophages are downregulated for the production of TLR-inducible cytokines including IL-1, IL-6, IL-8, TNF*α*, and IL-10 irrespective of the stimulus. This inability is associated with the markedly reduced MyD88, Toll/TIR-domain containing adaptor-inducing IFN*β* (TRIF) adaptor protein and TRAF-6 which results in NF*κ*B inactivation. However, in the mucosa of people with inflammatory bowel disease, intestinal macrophages may express high levels of NF*κ*B binding activity, and it is thought that these cells are newly recruited monocytes that have not been downregulated. Consistent with the observation in DCs, the PI3-K/Akt pathway in monocytes also suppresses both Map kinases and NF*κ*B in response to LPS resulting in decreased production of TNF*α* [38].

 Studies on PI3-K knockout mice support the idea that PI3-K negatively regulates TLR activation, as signaling by TLR2, 4, 5, and 9 is elevated in p85*α* deficient mice and LPS-induced IL-12 secretion is elevated in p110*β* deficient macrophages [39]. PI3-K appears to inhibit proinflammatory cytokine production *via *GSK3, a serine threonine kinase that inhibits the activity of Cyclin D1, *β*catenin, cjun and Myc *via* phosphorylation of specific residues [40]. PI3-K activation in response to TLR stimulation leads to the inhibition of GSK3 resulting in increased IL-10 production *via* CREB and its coactivator CBP binding (Figure 2(a)). GSK3 also inhibits AP-1 DNA binding which could also affect IL-10 expression. At the same time IL-12 is decreased due to less NF*κ*B activation because of competition for the CBP coactivator. Phosphoinositide-dependent kinase 1 (PDK1) is an important signaling component in the PI3-K pathway. Primary macrophages derived from mice with conditional knockout of PDK1 in myeloid lineages have elevated TNF*α* and IL-6 mRNA and release. While immediate TLR4 signaling is intact, these macrophages exhibit prolonged ubiquitination of TRAF-6 in response to LPS revealing a PDK-1 dependent negative feedback inhibition on NF*κ*B activation in macrophages [41]. Several phosphatases that regulate PI3-K, that is, PTEN, SHP-1, and Mapk phosphatase (MKP), have been investigated in the mechanism of the anti-inflammatory function of PI3-K in macrophages [42]. PTEN-deficient macrophages which have elevated PI3-K, showed reduced inflammatory cytokine, TNF*α*, and IL-6 production which was accompanied by reduced MAPK activation associated with increases in the Map kinase phosphatase, dual specificity phosphatase 1 (DUSP1), and increases in anti-inflammatory IL-10. DUSPs dephosphorylate p-Thr as well as pSer/p-Tyr sites on Map kinases [42]. The protein tyrosine phosphatase SHP-1 has also been shown to down regulate TLR-induced IL-12p40 production in macrophages through inhibition of PI3-K [43].

 Other reports demonstrate a pro-inflammatory role for PI3-K in monocytes *via* NF*κ*B activation, likely *via* the phosphorylation of p65 [26]. Evidence for both pro-inflammatory and proapoptotic signaling in response to TLRs in macrophages is emerging. TLRs, *via *the adaptor molecule, TLR interacting adaptor protein inducing IFN-*β* (TRIF) can act as death receptors with inflammatory and apoptotic pathways acting in parallel, where the final outcome depends on the magnitude of the responses [44].

### 4.2. Role of PI-3 Kinase Signaling in Intestinal T-Cells

Lamina propria T (LPT) cells are poor responders towards antigen-receptor triggering with very few T-cells proliferating in response to TCR/CD3-directed stimuli [45] (Figure 2(b)). T-cell activation *via* CD58/CD2 or B7/CD28 contributes to the accumulation of T-helper cells, increased T-cell proliferation and reduced apoptosis, all characteristic of inflammatory bowel disease. The first *in vivo* evidence of the proliferative hyporesponsiveness of LPT cells is an *in vivo* study in rats demonstrating both antigen-receptor-dependent and independent activation pathway downregulation [46]. Much lower T-cell proliferation was observed after *α*/*β*TCR stimulation with monoclonal antibody (mAb) compared to dual stimulation with anti CD2 and anti-CD28 mAb, and no proliferation was observed with anti-CD2 mAb alone. Hyporesponsiveness is restricted to the mucosa and cannot be found in the mesenteric lymph nodes or Peyer's patches. Work by Kamanaka's group explains the hypo-responsiveness of LPT cells. They showed that *α*/*β*TCR stimulation induces Foxp3+ regulatory T-cells (Treg) with high IL-10 production. Since these Tregs are anergic and suppressive, this likely explains the hypo-responsiveness [21]. 

#### 4.2.1. T-Cell Receptor and Costimulatory Signals

In contrast to antigen-presenting cells, T-cells employ PI3-K to promote inflammatory responses and proliferative responses such as IL-2 and IFN*γ* synthesis, downstream of co-stimulatory molecules such as CD28 (Figure 2(b)). PI3-K and NF*κ*B activation is necessary to mediate CD28-mediated proliferative responses in CD4+ T-cells. *In vitro* studies using human LPT cells have shown that LPT cells respond vigorously when stimulated through the CD2 receptor. CD2 stimulation represents an alternative mode of T-cell activation in LPT [47]. When compared to peripheral blood T-cells (PBT), LPT cells show an increased activation of the PI3-K/AKT/GSK-3*β* pathway in response to CD2 stimulation resulting in enhanced CD2-induced cytokine production in LPT, that is IL-2, TNF*α* and IFN*γ*, GMCSF, and CD40L. They also produce enhanced levels of IL-10 [48]. Although the T-cell population in the LP is almost exclusively CD45RO+, there were no significant differences in CD2 activation of PI3-K pathway in the total T-cell population of PBTs compared to PBT CD45RO+ T-cells [49]. Thioredoxin, a thiol disulfide oxidoreductase, is highly expressed in LPT and has been shown to inactivate the lipid phosphatase PTEN, and this may account for some of the increased CD2 responsiveness in these cells [50]. AKT-dependent regulation of NF*κ*B or nuclear retention of NFAT due to GSK3*β* inhibition may contribute to the increased cytokine production in response to CD2 stimulation in LPT. Increases in PI3-K-mediated signaling in response to CD2 stimulation may also be associated with increases in proliferation, as a recent study reported that the cell doubling time of LPT following CD2 stimulation is significantly shorter than that of PBT, and this was associated with increased, Rb phosphorylation [51]. Interestingly Rb phosphorylation is influenced negatively by inhibition of PI3-Kinase in T lymphocytes [52].

#### 4.2.2. TLR Signaling

An anti-inflammatory role for PI3-K signaling downstream of TLRs in intestinal T-cells has been reported. While TLR-signaling pathways in T-cells are poorly characterized, it has been demonstrated that in CD4+ T-cells, that CpGDNA (TLR9 ligand) stimulation of PI3-K/AKT which inhibits GSK3, attenuates excessive pro-inflammatory TLR9-mediated immune responses. GSK3*β* promoted the production of pro-inflammatory cytokines in primary murine and human intestinal T-cells while lowering secretion of the anti-inflammatory IL-10 by differential regulation of NF*κ*B and CREB activities [53]. The mechanism is likely similar to that described in innate immune cells (Figure 2(a)), where *in vivo *blockade of GSK3*β* lowered NF*κ*B activity with increased CREB DNA binding in intestinal lymphocytes from inflamed intestine. As CREB is a critical component for IL-10 production, inhibition of its DNA binding impairs IL-10 production. Notably, the inhibition of GSK3*β* did not alter TLR-induced immune responses of cells from a noninflamed microenvironment, while excessive pro-inflammatory reactions of cells from inflamed tissue were selectively reduced which suggests that inhibition of GSK3 could be used to lower exaggerated inflammatory responses in IBD.

It has also been demonstrated that in CD4+ T-cells, CpGDNA stimulation (TLR9 ligand) directly enhances proliferation, prevents anergy and augments humoral responses to a T-cell-dependent antigen by a MyD88 and PI3-K-dependent pathway. Mutation of Y257 in the SH2-containing TIR domain of MyD88 abrogated p85 binding, phosphorylation of AKT and GSK3 and IL-2 production as well as CpG DNA driven co-stimulatory proliferative responses to suboptimal concentrations of CD3 mAb [54]. The MyD88 death domain on the other hand was required for NF*κ*B activation and survival.

### 4.3. Role of PI3-K Signaling in Intestinal Epithelial Cells

#### 4.3.1. IL-1R Signaling

Normal epithelial cells express only 3 out of 4 of the p110 isoforms of PI3-K (*α*, *β* and *δ*), and p110*δ* is absent from Caco-2 cells, a widely used model of polarized epithelium. While all the subunits appear to catalyze the same enzymatic reactions, there are different cellular responses associated with them which may be due to different localizations or even nonenzymatic activities.

Intestinal epithelial cells from both IBD and normal controls have receptors for IL-1, IL-6, and GM-CSF, but not for TNF*α*, although they have been detected on adenocarcinoma cell lines. Caco-2 cells, an epithelial adenocarcinoma cell line, have receptors for IL-6 at both poles and for IL-1 at the basolateral surface and to a lesser extent at the apical pole (Figure 2(c)). T84 another intestinal adenocarcinoma cell line has receptors for IL-6 and IL-1 only at the basolateral pole. Functionally, IL-1 receptors enhance intestinal epithelial cell growth and have also been shown to enhance the growth of Caco-2 cells [55, 56]. Receptor density is greater on surface versus crypt epithelial cells [55].

Although IL-1*α* is constitutively expressed by epithelial cells, the expression of the pro-form of IL-1*β* is induced by NF*κ*B and later processed to the active form. Interleukin-1*β* and the type 1 IL-1R have been implicated in protection and control against several enteric pathogens including *Staphylococcus aureus, Salmonella enteric,* and *Shigella flexneri* as well as chemical-induced colitis [57–59]. IL-1R signaling protects mice from the attaching and effacing pathogen *Citrobacter rodentium*. Upon infection, mice lacking the type 1 IL-1R demonstrate increased mortality and severe colitis. It is thought that the protective effects against this pathogen might be mediated by the constitutive IL-1*αvia* a MyD88-dependent pathway. IL-1R^−/−^ mice fail to produce IL-6 and IFN*γ* [60]. It is not known if the protective effects of IL-1 are mediated by PI3-K. However, inhibition of PI3-K resulted in increased chloride secretion and barrier dysfunction suggesting that agonists that induce PI3-K may protect epithelial cells from immune-mediated apoptosis as well as function to limit chloride secretory diarrhea [61].

Intestinal epithelial cells are capable of inducing an acute phase response similar to hepatoma cells [62]. Intestinal epithelial cells produce IL-6 in response to IL-1. IL-6 leads to increased protective acute phase responses following tissue damage or infection [63]. In the Caco-2 cell line a PI3-kinase-dependent role for IL-1 induction of IL-6 gene transcription was reported [22]. This involved a PI3-K/AKT-dependent pathway upstream of the transcription factor activator protein 1 (AP-1) (Figure 2(c)). This pathway involved a kinase in the IKK complex, IKK*α*, which is phosphorylated by AKT on Thr23 upstream of AP-1. This is likely independent of the canonical AP-1 pathway *via* JNK activation and suggests that there is an alternative AP-1 activation pathway in intestinal epithelial cells (unpublished data). It is likely that this IL-1-induced pathway-mediating IL-6 transcription could also mediate the protective effects of IL-1 and that NF*κ*B may be involved in mediating more acute increases in IL-6 in immune cell types.

#### 4.3.2. TLR Signaling

Most intestinal epithelial cells are potently responsive to flagellin the specific ligand for TLR5 and hypo-responsive to TLR4 (LPS receptor). TLR4 deficiency makes the mouse susceptible to Dextran sulfate-induced colitis and feeding LPS to normal mice provides protection against DSS-induced colitis. This suggests that TLR4 activation by LPS may provide beneficial effects such as promoting epithelial cell proliferation and enhanced wound healing at the intestinal epithelium [64]. Despite the hypo-responsiveness of TLR4 in various intestinal epithelial cells, uncontrolled TLR4 activation is associated with necrotizing enterocolitis. Preterm infants show a higher expression of TLR4 in the intestine than normal infants rendering preterm infants highly susceptible to inflammation due to TLR4 activation by enteric microbes [65].

TLR4 signaling has been shown to exacerbate *Citrobacter. rodentium* infection. Both bacterial LPS and infection with *C. rodentium* inactivate Foxo3*α* in intestinal epithelia *in vivo* and *in vitro *[66, 67]. Foxo3 belongs to the family of tumor suppressor family of Forkhead transcription factors. It is located in the nucleus and regulates genes involved in cell cycle, apoptosis, and metabolism. Phosphorylation of Foxo is mediated by PI3-K as well as by IKK. Translocation to the cytoplasm by 14-3-3-mediated nuclear export, together with proteasomal degradation, mediates its inactivation [68, 69]. LPS and TNF*α*-mediated Foxo inactivation in HT-29 cells was controlled by the PI3-K pathway. Blocking PI3-K leads to attenuation of LPS and TNF*α*-induced IL-8 secretion in HT-29 cells and LPS induced IL-8 is increased in HT-29 cells, an intestinal epithelial adenocarcinoma cell line with silenced Foxo3*α* [66, 67]. IL-8 is a pro-inflammatory chemokine that is a chemo-attractant for neutrophils and lymphocytes. LPS was associated with down regulating the NF*κ*B inhibitor, IkB*α*, and in the case of TNF*α*, IKK was also involved in the pathway. It was also shown that Foxo3 localization in the cytosol and Foxo deficiency lead to severe intestinal inflammation *in vivo* in a Foxo3-deficient mouse. Foxo3-deficient mice develop more severe inflammatory responses to DSS compared to wild type mice [66].

TLR5 activation is also associated with IBD [70]. It has been suggested that activation of different isoforms of PI3-K may explain the differential outcomes on TLR5 activation in epithelial cells.

TLR5 is localized on the basolateral side of epithelial mucosa, and responsiveness is therefore increased with impaired barrier function as in IBD. Inhibition of PI3-K with wortmannin or LY204002 increased both IL-6 and IL-8 production in response to flagellin in T84 cells [71]. Systemic cytokine release in response to intraperitoneal injections of flagellin in p85^−/−^ mice was significantly higher compared to heterozygous littermates. Another study in T84 cells demonstrated a PI3-K dependent anti inflammatory pathway activated by Salmonella [72]. In this study, inhibition of PI3-K in T84 cells resulted in increased IL-8 production. Contrary to these 2 studies, a paper by Sang et al., (2006) demonstrated that inhibition of PI3-K using dominant negative p85, Akt or LY294002 reduced IL-8 production in response to flagellin indicating that PI3-K augments flagellin-mediated inflammatory responses in intestinal epithelial cells [73]. Zeng et al. 2006 showed that flagellin induces a pro-inflammatory cascade, and in the absence of NF*κ*B or PI3-K/Akt signaling, apoptosis is initiated in parallel [74].

## 5. Effect of PI3-K Inhibition in Mouse Models of Inflammatory Bowel Disease

### 5.1. Effect of PI3-K*γ* Inhibition in Dextran Sulphate Sodium (DSS) and 2,4,6-Trinitrobenzenesulphonic Acid (TNBS) Mouse Models of Intestinal Inflammation

The role of PI3-K in mouse models of IBD is beginning to emerge. Using specific pharmacological inhibitors of PI3-K*γ*, attenuation of DSS-induced colitis was demonstrated [75]. The inhibitor, AS605240, was administered starting on the same day as DSS administration in the acute colitis model and on day 11 after DSS administration in the chronic colitis model (Figure 1(b)). AS605240 had protective and therapeutic effects in both acute and chronic DSS colitis *in vivo* and significantly decreased the clinical and histopathological symptoms of DSS-fed mice and increased survival in the acute model. This was accompanied by decreases in phosphorylated Akt in immunological cells in both inflamed colon and spleen of DSS-fed mice and decreases in macrophage together with neutrophil and CD4+ T-cell infiltration. Additionally, levels of the pro-inflammatory IL-1*β*, TNF*α* and IFN*γ* in the colon was decreased by AS605240 with accompanying restored levels of the anti-inflammatory cytokine IL-4.

Another study of the effects of PI3-K*γ* on acute DSS colitis was done using PI3-K*γ* mutant mice harboring a kinase dead form of this PI3-K isoform [76]. Both clinical and histopathological parameters showed that severity of colitis was significantly reduced in PI3-K*γ*-kinase inactive mice compared to controls. This was accompanied by significantly more pro-inflammatory Th1 cytokines such as IL-12, TNF*α*, and IFN*γ* and more IL-10, suggesting a role for PI3-K*γ* in the negative regulation of these cytokines. Increased numbers of resident macrophages and T-cells in the colonic lamina propria in the unstressed condition were also observed, suggesting that PI3-K*γ* may not only play a role in leukocyte recruitment in response to injury and inflammation but also regulate emigration of leukocytes from the lamina propria under physiological conditions. The failure to recruit new leukocytes to the mucosa upon DSS treatment of the mice suggests that PI3-K*γ* functions in lamina propria leukocyte trafficking.

Another study using PI3-K*γ* knockout mice in which the isoform is absent, were treated with DSS [77]. This is an important difference as PI3K*γ* also has a kinase-independent role as a scaffold protein. Similar to the results above, absence of a functional PI3-K*γ* protects mice from DSS-induced colitis and the knock out mice fail to recruit T-cells and macrophages to the colon after DSS treatment. One of the major differences with the previous study is that they observed a decrease in TNF*α* production in the PI3-K*γ* knock-out mice upon treatment with DSS. Because a mouse bearing a point mutation in the kinase domain, making the PI3K*γ* kinase dead was used, this might resemble the effects similar to small-molecule inhibition. Thus, absence of kinase activity in the PI3-K*γ* kinase inactive mouse might be responsible for the observed increases in TNF*α*. This study demonstrated that PI3-K*γ* deficient mice, in addition to having less colonic inflammation also had a lower incidence of colitis-associated tumors. These studies all compliment a prior study showing that intravenous administration of small interfering RNAs against p85*α* attenuates inflammation in a DSS mouse model of colitis [78]. The fact that increases in AKT phosphorylation are observed in intestinal mucosa of patients with ulcerative colitis suggests that inhibition of this pathway may prove efficacious in the treatment of the disease in humans [79].

More recently, it has been reported that the PI3-K*γ* inhibitor, AS605240, ameliorates TNBS induced colitis in mice by affecting the functional activity of Treg cells, CD4+CD25+FoxP3+ cells [80]. The TNBS model of IBD has an elevated Th1 response with macrophages producing large amounts of IL-12, IFN*γ*, and IL-1 and is a model more for Crohn's disease. Oral administration of the drug reduced colonic expression of IL-1*β*, the chemokine, CXCL-1/KC, macrophage inflammatory protein, MIP-2, and TNF*α* in an NF*κ*B-dependent manner. Phosphorylation of the p65 subunit of NF*κ*B significantly decreased in colon tissue. Increases in CD25 FoxP3 and IL-10 expression were observed in isolated lamina propria of AS605240-treated mice which coincided with increased percentages of Treg CD4+CD25+FoxP3+ cells. Thus, these results suggest that AS605240 has multiple inflammatory targets *via* NF*κ*B inhibition, while increasing the numbers of anti-inflammatory Treg cells.

Another PI3-K inhibitor PIK-75 which inhibits both the *α* and *γ* isoforms has also been shown to attenuate DSS-induced colitis by suppressing the production of pro-inflammatory mediators in an NF*κ*B-dependent manner as well as reducing the inflammatory cellular infiltrate into the colonic interstitium [78]. Given the fact that PIK-75 is known to exhibit anticancer activity, this study reinforces the cross-therapeutic functionality of potential drugs. PIK-75 potently inhibits *in vitro* LPS-induced production of TNF*α* and IL-6 from freshly isolated human monocytes with corresponding inhibition of NF*κ*B. Interestingly however, PIK-75 under *in vitro* conditions markedly inhibited the production of IL-10 from human PBMCs stimulated with a combination of anti-CD3 and anti-CD28 MAbs. RT-PCR analysis also demonstrated that PIK-75 reduced IL-10 mRNA in the colon of DSS-treated mice.

## 6. PI3-Kinase Inhibition in Inflammation-Induced Colorectal Cancer

The PI-3K pathway has been shown to play an important role in the regulation of intestinal epithelial proliferation, survival and wound healing. It will therefore be important to address the role of each isoform in both normal cellular homeostasis and in disease before using isoform specific inhibitors clinically [81]. Each isoform is capable of regulating multiple cellular functions but with significant redundancy which may also limit the clinical use of isoform specific inhibitors.

Class 1A and class 3 PI3-kinases are strongly expressed in colonic epithelial carcinoma cell lines, and there is increased PI3-kinase activity in colorectal carcinoma specimens. Both p110*α* and p110*β* play important roles in human colon cancer growth: p110*β* has a specific role in *de novo *DNA synthesis, and p110*α* determines cell survival [82]. The transforming functions of PI3-K*γ* in colon carcinoma are linked to disruption of intercellular adhesion and myeloid cell invasion [83].

There are potentially multiple mechanisms for PI-3 kinase constitutive activation in colon cancer, for example, direct PI-3-K activation through PIK3CA mutation, PTEN loss, activation of AKT itself through activating mutations in its PH domain, receptor tyrosine kinases such as ERBB3 activation as well as KRAS (which is mutated in up to 45% of colorectal cancers) and which is upstream of both the PI-3 kinase and Map kinase pathways [84]. Some colorectal tumors are mutated in more than one of these pathways. Therefore, the success of PI3-K inhibitors alone will depend on the type of mutation manifested in the patient. It is likely that a more targeted and personalized medicine approach will be required for success, with specific PI3-K inhibition used in conjunction with conventional cytotoxic therapies. A positive predictor of response may be detection of activating mutations in the PI-3-K gene itself, while KRAS mutations would likely be a negative predictor of response. It has recently been shown that receptor tyrosine kinases have control of PI-3K signaling in human KRAS mutant colorectal cancers and PI3-K may be involved in maintenance of the tumor phenotype after transformation. Infact only about 7% of patients in a recent study were reported to have a PIK3CA mutation without a KRAS mutation. The percent of patients that might benefit from PI3-kinase inhibitors may increase when more is known about PTEN regulation in these cancers [85, 86].

Concern from the initial first generation PI3-K inhibitors was that the second generation inhibitors might be toxic in humans was unwarranted. Third generation inhibitors in preclinical models are showing promise as anti-cancer therapeutics. The importance of PI3-K downstream of insulin signaling was a further concern; however, in early clinical evaluation of the inhibitors the only effect has been a rise in insulin. Several inhibitors of PI3-K pathway are currently in clinical development for colorectal cancer and have been shown to potentiate the effects of cytotoxic therapy. This is likely because PI3-K pathway mediates tumor survival following cytotoxic therapy. Perifosine, in phase 11 clinical trials, is an inhibitor of AKT and has shown some promise in combination with other inhibitors. MK-2206, also an AKT inhibitor, has recently completed phase 1 study. The reader is referred to a recent paper of these and other PI3-K pathway inhibitors currently being tested in colorectal cancer [85].

## 7. Conclusions and Future Studies

The intestinal mucosa has adapted an immune system to respond appropriately to commensal and pathogenic bacteria to maintain immune homeostasis. The PI3-kinase signaling pathway downstream of TLRs, TCR, and co-stimulatory receptors is an important mediator of this immune homeostasis. Dysregulation of this pathway in innate and adaptive immune cells and in the intestinal epithelium can lead to inflammatory disorders including inflammatory bowel disease and its associated cancers. Great strides have been made in the development of isoform specific PI3-K inhibitors and have lead to the identification of PI3-K*γ* as an important isoform in intestinal inflammation; it will be necessary to test the efficacy of these inhibitors in terms of their future therapeutic use in humans.

## Abbreviations

(PI3-K): Phosphatidylinositol 3-kinases
PIP: Phosphatidylinositol(3)
PIP2: Phosphatidylinositol(3,4)
PIP3: Phosphatidylinositol(3,4,5).
